# Whey Beverage Emulsified System as Carrying Matrix of Fennel Seed Extract Obtained by Supercritical CO_2_ Extraction: Impact of Thermosonication Processing and Addition of Prebiotic Fibers

**DOI:** 10.3390/foods11091332

**Published:** 2022-05-03

**Authors:** Adela Cristina Martinez Urango, Maria Isabel Landim Neves, Maria Angela A. Meireles, Eric Keven Silva

**Affiliations:** School of Food Engineering, University of Campinas, Rua Monteiro Lobato, 80, Campinas 13083-862, SP, Brazil; adelmartinez08@gmail.com (A.C.M.U.); isabellandimneves@gmail.com (M.I.L.N.); maameireles@lasefi.com (M.A.A.M.)

**Keywords:** whey protein, high-intensity ultrasound, xylooligosaccharides, browning index, dairy beverage

## Abstract

Whey beverages that were enriched with fructooligosaccharides (FOS) and xylooligosaccharides (XOS) were used for carrying *Foeniculum vulgare* extract that was obtained by the supercritical CO_2_ extraction technique to produce novel functional products. Fennel-based whey beverages were subjected to thermosonication processing (100, 200, and 300 W at 60 °C for 15 min) to verify the performance of the dairy colloidal system for protecting the bioactive fennel compounds. The impacts of thermosonication processing on the quality attributes of the functional whey beverages were examined according to their droplet size distribution, microstructure, kinetic stability, color parameters, browning index, total phenolic content (TPC), and antioxidant capacity by DPPH (2,2-diphenyl-1-picrylhydrazyl) and ABTS (2,2-Azino-bis-(3-ethylbenzothiazoline)-6-sulphonic acid) assays. The enrichment of the whey beverages with FOS and XOS did not affect their kinetic stability. However, the addition of prebiotic dietary fibers contributed to reducing the mean droplet size due to the formation of whey protein–FOS/XOS conjugates. The thermosonication treatments did not promote color changes that were discernible to the human eye. On the other hand, the thermosonication processing reduced the kinetic stability of the beverages. Overall, the colloidal dairy systems preserved the antioxidant capacity of the fennel seed extract, regardless of thermosonication treatment intensity. The whey beverages enriched with FOS and XOS proved to be effective carrying matrices for protecting the lipophilic bioactive fennel compounds.

## 1. Introduction

Infusion is a popular technique to produce beverages known as tea from the steeping of aromatic, medicinal, and spice plants with hot water. These beverages that are prepared by infusing the leaves, flowers, and roots of plants are aqueous extracts that are rich in hydrophilic bioactive compounds. The term “milk tea” refers to several forms of beverage that contain some combination of tea and milk. It is worldwide enjoyed as both a hot and cold beverage, mainly in China, Japan, Indonesia, and North Korea. However, some countries have created their versions of these beverages, which have increased their popularity in America and Europe in the last decades [1,2]. Milk has a fundamental role in the sensory properties of the infusion. Its colloidal system provides a smoother flavor and slightly sweetens the infusion, decreasing the astringent and bitter flavor. Milk compounds bind to some bitter chemical compounds, covering up undesirable flavors [3]. Many health benefits can be associated with milk tea consumption due to its plant material that is rich in phytochemical compounds with antioxidant properties, which provides health benefits. On the other hand, milk is a nutritious food that contains essential nutrients, including calciferol (vitamin D), cobalamin (vitamin B12), proteins, calcium, and potassium [4,5,6].

In this context, new alternatives to manufacture medicinal plant-enriched beverages are needed to provide a wide variety of products to the consumer market. Whey is broadly used for the formulation of dairy products known as whey beverages. It presents several health benefits due to its high content of essential amino acids, proteins, antioxidant compounds, and immunostimulants [7,8]. From an economic point of view, whey-based beverages are products with lower production costs due to the use of a by-product of the cheese industry. Furthermore, whey proteins present emulsifying properties that are used for the kinetic stabilization of oil-in-water emulsions [9,10]. In this sense, whey beverages are promising dairy systems that are able to encapsulate and carry lipophilic bioactive compounds extracted from plant matrices and are recognized for their health-promoting properties [11]. 

Among aromatic, medicinal, and spice plants, *Foeniculum vulgare* is a potential plant material for developing whey beverages due to its sensory attributes such as flavor and aroma. It is an aromatic herb with condiment properties that is popularly known as fennel and is used in folk medicine due to its carminative, antifungal, and antibacterial properties [12,13]. Fennel seeds have a high antioxidant capacity that is associated with its volatile compounds, such as anethole, fenchone, estragole (methyl-chavicol), α-phellandrene, and myrcene [14,15]. Thus, the development of novel dairy beverages based on lipophilic bioactive compounds from fennel seeds and whey could provide therapeutic food products [16]. 

One of the biggest trends for the dairy beverage market is manufacturing whey beverage enriched with prebiotic carbohydrates [17,18]. Prebiotic compounds can modulate gut microbiota, improving human immune function according to their “fertilizer” effects for the growth of probiotic microorganisms [19]. The use of fructans such as fructooligosaccharides (FOS) and inulins with different degrees of polymerization have been evaluated as smart strategies to increase the health benefits obtained by the consumption of dairy products [20]. Fermentable carbohydrates such as galactooligosaccharides (GOS), isomaltooligosaccharides (IMOS), and xylooligosaccharides (XOS) have emerged as ingredients for the design of functional food products [21]. Likewise, innovative technologies applied to the processing of dairy beverages have been investigated for providing alternative stabilization treatments based on non-thermal or mild thermal processes to inactivate pathogenic and spoilage microorganisms and endogenous enzymes [22]. Conventional heat treatments employing high temperatures, such as sterilization (≥121 °C), may promote the degradation of thermolabile compounds, mainly vitamins [23]. On the other hand, there is a growing demand for the consumption of non-thermal-treated products due to the physicochemical, rheological, and sensorial deterioration observed after high-energy processing based on thermal treatments [24]. In this way, an interesting emerging technology for the stabilization of dairy products is the thermosonication treatment. The combined effect of mild or moderate heat treatments with acoustic energy provided by low-frequency ultrasound technology has been highlighted as a powerful sterilization technique of food and beverages [25]. This innovative technique’s main advantage is to promote an effective spore inactivation in dairy products with a lower temperature compared to thermal processing alone at the same temperature conditions [26]. Thus, thermosonication treatments can ensure consumers’ safety while simultaneously preserving food quality attributes.

Many studies have reported the performance of the thermosonication treatment as a promising stabilization technique for the inactivation of foodborne pathogens, spoilage microorganisms, and enzymes [27,28,29,30]. Regarding dairy products, thermosonication has been recognized as a more effective technique to reduce the spore load of these products than thermal processing alone operating at the same temperature [31]. Parreiras, et al. [32] thermosonicated human milk using ultrasonic bath equipment at 40 kHz and a nominal power of 100 W. *Staphylococcus aureus*, *Escherichia coli*, and *Salmonella* spp. had their load reduced 5.6, 5.4, and 3.7 log, respectively, after a thermosonication treatment at 60 °C for 4 min. Thus, this study focused on sweeping thermosonication process conditions to understand the impact of this innovative technology on the quality attributes of whey beverages enriched with prebiotic ingredients regarding their physical, chemical, and functional properties. Although several studies have investigated the positive effects of thermosonication on the microbial quality of dairy products such as skim milk [31,33], fermented milk drink [34], goat milk [35], human milk [32], and others, studies focused on the technological, chemical, and functional properties of thermosonicated dairy beverages are still scarce.

Therefore, this work aimed to evaluate the whey beverage colloidal system as a carrying matrix of lipophilic bioactive compounds from fennel seeds obtained by the supercritical CO_2_ extraction technique to produce a novel functional dairy product. Fennel-based whey beverages were enriched with prebiotic fibers (FOS and XOS) and subjected to thermosonication processing (100, 200, and 300 W at 60 °C for 15 min) to verify the performance of these dairy colloidal systems for protecting the bioactive fennel compounds. Moreover, we monitored the thermosonication treatment temperature to clarify how the combined mild heat treatment and acoustic energy processing could affect the quality aspects of the functional beverages.

## 2. Materials and Methods

### 2.1. Ingredients, Chemicals, and Reagents

The food ingredients used to produce whey beverages were skimmed milk powder (Molico^®^, São Paulo, Brazil), whey powder (Alibra^®^, São Paulo, Brazil), PreticX^TM^ from non-genetically modified corn (XOS, DP = 2 to 6) (AIDP Inc., City of Industry, CA, USA), and Orafti^®^ FOS from chicory (FOS, DP = 5) (Sweetmix, Sorocaba, Brazil). Carbon dioxide (CO_2_; purity > 99.8%) was supplied by White Martins (São Paulo, Brazil). Folin–Ciocalteu reagent, sodium carbonate, ethanol, 2,2-diphenyl-1-picrylhydrazyl (DPPH^•^), Trolox (6-hydroxy-2,5,7,8-tetramethyl chroman-2-carboxylic acid), potassium persulfate, 2,2′-azinobis-(3-ethylbenzothiazoline-6-sulfonic acid)-(ABTS^•+^), and gallic acid were purchased from Sinergia-Científica (Campinas, Brazil). 

### 2.2. Supercritical CO_2_ Extraction of Lipophilic Bioactive Compounds from Fennel Seeds

**Figure 1:**
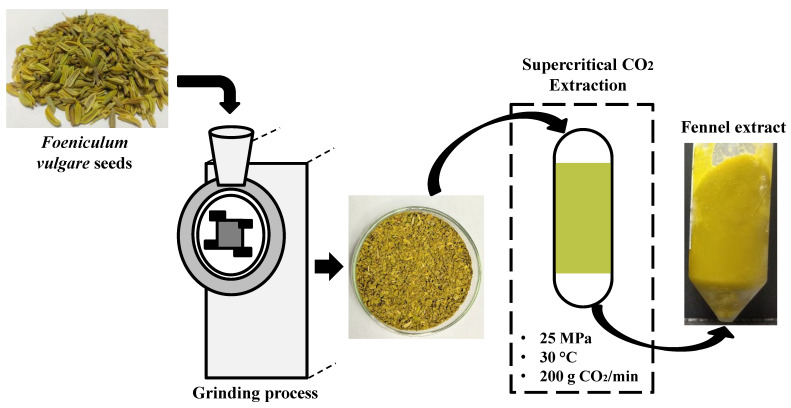
Supercritical CO_2_ extraction of bioactive compounds from fennel seeds.

Figure 1 illustrates the experimental procedures for obtaining the lipophilic bioactive compounds from fennel. The fennel seeds were acquired in the Mercado Municipal de Campinas (São Paulo, Brazil). The seeds were ground in a model MA-340 knife mill (Marconi, Piracicaba, Brazil). Afterward, the particle size distribution was determined in a model 1868 vibratory system (Bertel, Caieiras, Brazil) using sieves from 9 to 80 mesh (Tyler series, Wheeling, WV, USA). The mean particle diameter of the fennel seeds was 0.97 mm. The ground particles were stored at −18 °C until the extraction assays.

Fennel extracts were obtained by the supercritical fluid extraction (SFE) technique using pilot scale SFE equipment (Thar Technologies, Pittsburgh, PA, USA) with two identical 5-L extractors (SFE 2 × 5 L) employing a CO_2_ flow rate of 200 g/min. The solvent (S) to feed (F) ratio (S/F) was equal to 20. The extractions were performed at 30 °C and 25 MPa. The temperature and pressure conditions were optimized according to Moura et al. [14]. The extracts were collected in glass flasks and stored at −18 °C until their use in the whey beverage formulation step.

### 2.3. Manufacturing Fennel-Based Whey Beverages

Table 1 presents the formulation of the fennel-based whey beverages prepared from fennel extracts and prebiotic carbohydrates. Three whey beverages were formulated for evaluating the influence of the addition of FOS or XOS on their technological and functional properties. A control whey beverage, named standard, was prepared without prebiotic carbohydrates. 

**Figure 2:**
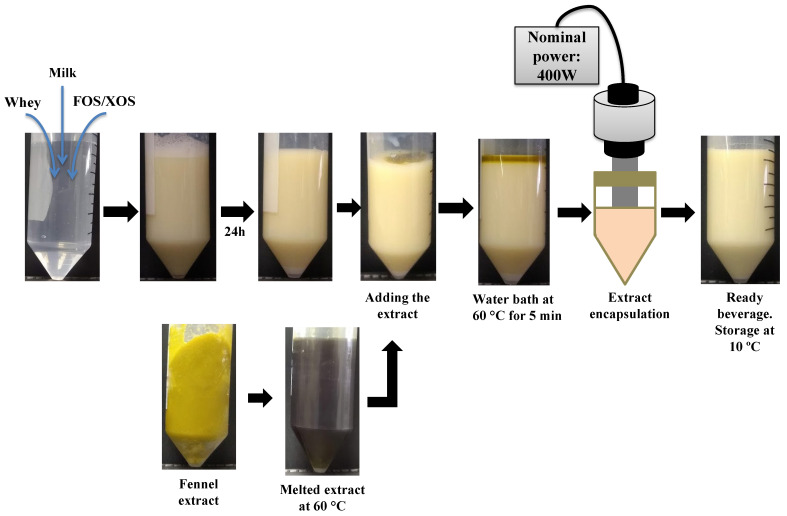
Manufacturing process of the fennel-based whey beverages.

The whey beverage production was divided into two stages: ingredients preparation and the encapsulation of fennel extract, as shown in Figure 2. Initially, the prebiotic fiber was dissolved in water. FOS was dissolved in hot water (70 °C) and cooled to room temperature. The other ingredients were then incorporated using a vortex mixer (Sinergia, São Paulo, Brazil). The suspensions were stored at room temperature for 24 h to ensure molecule saturation of the materials. The fennel extract was melted in a water bath at 60 °C and homogenized in a vortex for 6 s before its addition to the formulation. The mixture of ingredients was also placed in a water bath (60 °C) for 5 min, and the extract was incorporated into the beverage. The sample was sonicated at a nominal power of 400 W for 3 min to carry the fennel extract in the dairy system using a 13 mm ultrasonic probe at 19 kHz (Unique, Indaiatuba, Brazil). For this nominal power, the ultrasound equipment provided to the samples an acoustic power of 20 ± 1 W [36]. The encapsulation process was carried out at 25 °C. 

**Figure 3:**
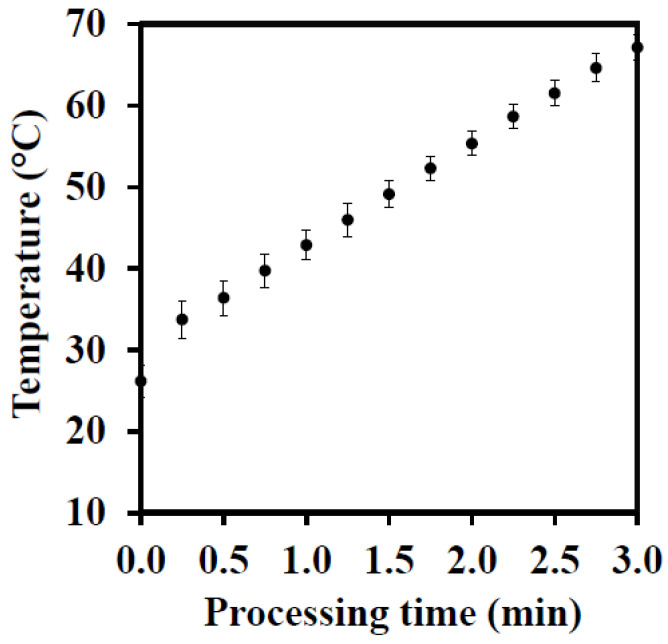
Thermal history of the ultrasound-assisted emulsification of fennel seed extract.

Figure 3 shows the thermal history of the samples during their ultrasound-assisted emulsification step. All whey beverages, standard, FOS, and XOS, exhibited the same thermal behavior. After the processing step, all samples were stored at 10 °C.

### 2.4. Thermosonication Treatments

The impact of the thermosonication treatment on the thermal history of whey beverages was evaluated by applying nominal powers of 100, 200, and 300 W, corresponding to specific energies of 3.6, 7.2, and 10.8 kJ/g, respectively, at 40, 50, and 60 °C for 15 min. The thermosonication treatments also were performed using a 13 mm ultrasonic probe at 19 kHz (Unique, Indaiatuba, Brazil). The acoustic powers provided for samples at the nominal powers of 100, 200, and 300 W were 4.6 ± 0.4, 8.5 ± 0.1, and 14.5 ± 0.3 W, respectively [36]. The ultrasound specific energy provided to the samples was calculated according to Equation (1) [37]. From the results observed in this previous step, the process temperature was fixed at 60 °C, and the effects of the different nominal powers on the technological and functional properties of the whey beverages were studied. Non-thermosonicated (NTS) beverages were used as control treatments. Thermosonication treatments were applied in a 50 mL Falcon tube with 25 g of whey beverage at 10 °C using a 250 mL double-walled beaker with water recirculation. The ultrasound probe was immersed into the Falcon tube center to a depth of 10 mm. After their processing, each sample was cooled in an ice bath until 25 °C or stored at −18 °C until further chemical analyses.

(1) $$Specific\;energy\left[\frac{J}{g}\right]=\frac{Nominal\;power\;\left(W\right)\times processing\;time\;\left(s\right)}{sample\;mass\;\left(g\right)}$$

### 2.5. Experimental Design

A full factorial experimental design (3 × 3) was used to evaluate the impact of the enrichment of whey beverages with different prebiotic fibers (standard, FOS, and XOS) and thermosonication treatments at different nominal powers (100, 200, and 300 W) on the technological, chemical, and functional properties of the fennel-based whey beverages. All experiments were performed in quadruplicate (*n* = 4). Therefore, thirty-six beverages were produced and examined.

### 2.6. Droplet Size Distribution

The droplet size distribution and mean diameter of the fennel-based whey beverages were determined at 25 °C by a light-scattering technique using a Mastersizer 3000 laser diffractor (Malvern Instruments, Malvern, UK). The mean droplet diameter was calculated based on the mean diameter of a sphere of similar area (superficial mean diameter -$D_{3,2}$, Equation (2)). The samples were analyzed by a wet method with dispersion in water and a refractive index of 1.52. The droplet distribution was expressed according to $d_{10}$, $d_{50}$, and $d_{90}$, which are the cumulative diameters at 10%, 50%, and 90%, respectively.

(2) $$D_{3,2}=\frac{\sum n_{i}d_{i}{}^{3}}{\sum n_{i}d_{i}{}^{2}}$$

where: $d_{i}$ is the mean diameter of the droplets and $n_{i}$ is the number of droplets.

### 2.7. Microstructure

The samples were poured onto microscope slides, covered with glass coverslips, and observed using a Model DMLM optical microscope (LEICA, Cambridge, UK) with the 200×, 500×, and 1000× objective lenses. The last one was performed using immersion oil.

### 2.8. Kinetic Stability

The kinetic stability of the samples was evaluated using a near-infrared backscattering profile technique. Each formulation was transferred to specific glass tubes and stored in the same condition mentioned before to measure the stability using a Turbiscan MA 2000 light backscatter scan analyzer (Formulaction, Toulouse, France). The measurements were performed immediately after sample production (0) and after 24, 48, and 72 h of cold storage at 4 °C. The Turbiscan Stability Index (TSI) was calculated as the sum of all destabilization processes that occur along the measuring cell according to Equation (3).

(3) $$TSI=\sum_{j}\left|scan_{ref}\left(h_{j}\right)-scan_{i}\left(h_{i}\right)\right|$$

where $scan_{ref}$ and $scan_{i}$ are the initial backscattering value and the backscattering value after 24, 48, and 72 h of storage, respectively; $h_{j}$ is the given height in the measuring cell and TSI is the sum of all the scan differences from the bottom to the top of the tube.

### 2.9. Color Parameters

The samples’ color parameters were determined by a CR-400 apparatus (Konica Minolta, Tokyo, Japan) using the CIELab scale ($L^{*}$, $a^{*}$, and $b^{*}$) with reflectance mode using illuminant D65 and an observation angle of 10°. The color difference (ΔE), chromaticity $\left(C^{*}\right)$, hue angle $\left(H^{*}\right)$, and browning index $\left(BI^{*}\right)$ were calculated according to Equations (4)–(7), respectively.

(4) $$\Delta E=\sqrt{{\left(L_{0}^{*}-L^{*}\right)}^{2}+{\left(a_{0}^{*}-a^{*}\right)}^{2}+{\left(b_{0}^{*}-b^{*}\right)}^{2}}$$

(5) $$C^{*}=\sqrt{a^{*}{}^{2\;}+b^{*}{}^{2\;}\;}$$

(6) H* =tan−1 (b*a*)

(7) $$BI^{*\;}=\frac{100\left(x-0.31\right)}{0.172}\;\;x=\frac{a^{*}+1.75L^{*}}{5.645L^{*}+a^{*}-3.012b^{*}}$$

where the subscript “0” refers to the color measurement of the non-thermosonicated beverages, $L^{*}$ represents lightness and darkness, $a^{*}$ represents red and green, and $b^{*}$ represents blue and yellow.

### 2.10. Chemical and Functional Properties

#### 2.10.1. Emulsion Break-Up Procedure

The lipophilic bioactive compounds from fennel seed extract carried in the beverages were released to proceed with the chemical and functional characterization. The whey beverages were thawed and homogenized using a vortex. Then, 2 mL of the sample was mixed with 8 mL of ethanol using a vortex for 3 min. The mixture was centrifugated at 160× *g* for 10 min, vortexed for 3 min, and placed for 10 min in an ultrasonic bath. The same procedure was performed two more times to ensure the complete extraction. The diluted extracts in ethanol (1:3 *v*/*v*) were collected and stored at −18 °C until for further characterization assays.

#### 2.10.2. Determination of Total Phenolic Content

The total phenolic content (TPC) was determined using the Folin–Ciocalteu colorimetric method according to Arruda et al. [38]. An aliquot of 300 µL of diluted extract in H_2_O (1:3 *v*/*v*), 300 µL of Folin–Ciocalteu reagent, and 2400 µL of sodium carbonate (5%, *w*/*v*) were mixed. Then, the reaction solution was kept in the dark for 20 min. The absorbance was measured at 760 nm against a blank using an 800XI UV-VIS spectrophotometer (FEMTO, São Paulo, Brazil). A calibration curve using gallic acid (10–90 μg/mL; R^2^ = 0.999) as the standard was employed to quantify the TPC. The results were expressed as µg gallic acid equivalents per milliliter of beverage (µg GAE/mL).

#### 2.10.3. In Vitro Antioxidant Capacity

Free radical scavenging activity was measured using the methodology reported by Brand-Williams et al. [39]. The experiments were performed on freshly prepared ethanolic solutions of DPPH^•^ (0.004% *w*/*v*). In brief, 800 µL of the extract was mixed with 4000 µL of DPPH^•^ solution using a vortex. After 30 min of the reaction, the absorbance of the remaining DPPH^•^ was measured at 517 nm on an 800XI UV-VIS spectrophotometer (FEMTO, São Paulo, Brazil). The antioxidant capacity was expressed as a percentage of the absorbance of the control DPPH^•^ solution, which was obtained from the following equation: %Activity = [(A_DPPH_ − A_sample_)/A_DPPH_)] × 100, where A_DPPH_ is the absorbance value of the control and A_sample_ is the absorbance value of the test solution. Trolox was used as a standard using a calibration curve (5–50 μg/mL, R² = 0.998), and the results were expressed as micromoles of Trolox equivalents per milliliter of whey beverage (μmol TE/mL). 

The ABTS^•+^ scavenging capacity assay was determined as described by Le et al. [40]. The method is based on the decolorization of the ABTS^•+^ radical cation to determine the antioxidant potential of the samples. The ABTS^•+^ radical cation solution was prepared in advance by reacting aqueous ABTS^•+^ solution (7 mM) with potassium persulfate (2.45 mM). In the analysis, diluted ABTS^•+^ solution with an absorbance of 0.70 ± 0.02 at 734 nm was employed. The assay was performed using a quartz cuvette. The reaction system was composed of 800 μL of diluted extract in ethanol (1:3 *v*/*v*) and 4000 μL of ABTS^•+^ solution, followed by a 6 min incubation at room temperature. The absorbance values were measured by an 800XI UV-VIS spectrophotometer (FEMTO, São Paulo, Brazil) at 734 nm in triplicate. The free radical scavenging activity was expressed as a percentage of the absorbance of the ABTS^•+^ control, which was obtained from the following equation: %Activity = [(A_ABTS_ − A_sample_)/A_ABTS_] × 100, where A_ABTS_ is the absorbance value of the ABTS^•+^ control and A_sample_ is the absorbance value of the test solution. A calibration curve was plotted from the absorbance reduction and concentration of the Trolox (5–50 μg/mL, R^2^ = 0.998). The results were expressed as micromoles of Trolox equivalents per milliliter of whey beverage (μmol TE/mL).

### 2.11. Statistical Analysis

Minitab 18^®^ software was used to verify the impact of the addition of prebiotic fibers and thermosonication process conditions on the physical, chemical, and functional properties of fennel-based whey beverages according to the proposed experimental design. Analyses of variance (ANOVA) at a significance level of 5% (*p*-value < 0.05) were performed.

## 3. Results and Discussion

### 3.1. Thermosonication Process Design 

**Figure 4:**
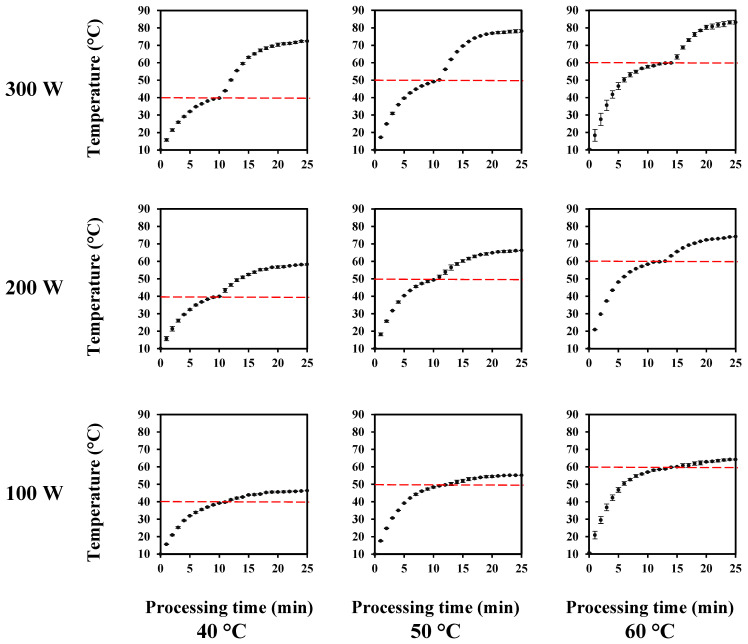
Thermal history of the whey beverages during their thermosonication treatment.

Figure 4 presents the thermal history of the fennel-based whey beverages during their thermosonication treatment. All of the whey beverages, standard, FOS, and XOS, presented the same thermal history due to their similar formulation. Thermal history describes how the beverage temperature changes as a function of the thermosonication treatment. All whey beverages were manufactured employing the acoustic energy provided by an ultrasonic probe for emulsifying the lipophilic bioactive compounds from fennel obtained by the supercritical CO_2_ extraction technique. In the emulsification step, the samples achieved up to 68 ± 1 °C according to their thermal history (Figure 3). 

For the thermosonication treatments, the initial temperature of the whey beverages was set to 10 °C. The holding time for thermosonication treatment refers to the heating time of the samples, starting from 10 °C, until reaching the working temperature (40, 50, and 60 °C), that is, the thermosonication temperature. Therefore, the holding time was the time for which the beverage was heated to reach the working temperature, and the processing time was the time that had elapsed since the beginning of ultrasound treatment. The red dotted line indicates the working temperature. In this regard, the crossing of the red dotted lines with experimental points indicates the beginning of the sonication treatment. The combined effect of the heat treatment and acoustic energy on the temperature increase of the whey beverages during their processing (Figure 4) demonstrated that the nominal power and working temperature had a strong impact on the temperature profile. Increasing the levels of both process variables increased the maximum temperature reached by the samples. The greater ΔT (maximum temperature - working temperature) values were observed for the thermosonication treatments applying 300 W. For this nominal power, ΔT values of 32, 28, and 24 °C were observed for working temperatures of 40, 50, and 60 °C, respectively. 

The acoustic cavitation phenomenon generated from the application of a low-frequency ultrasound in a liquid medium promotes the formation and subsequent collapse of microbubbles. These cavitation microbubbles release a high punctual amount of mechanical and thermal energy in the sonicated liquid medium, increasing its temperature during the processing time [41]. The increase in nominal power from 100 W to 300 W intensified the acoustic cavitation effects in the whey beverages, resulting in greater ΔT values. Wu et al. [42] reported that the increase in ultrasonic power from 7.98 W to 32 W decreased the microbubble collapse time, increasing the acoustic cavitation intensity. Thus, the combination of an external heat source and acoustic energy influenced the temperature profile. Therefore, the thermosonication process design for the processing of food products is a critical issue, mainly for those products rich in thermolabile compounds, such as vitamins and some phenolic compounds. Several studies on the thermosonication of foods only present the working temperature without mentioning the maximum temperature achieved for the samples during their processing. For example, for the working temperature of 40 °C, our results demonstrated that when increasing the nominal power from 100 W to 300 W, ΔT values from 4.2 to 32 °C were observed for the whey beverages. In other words, a thermosonication treatment presented with a working temperature of 40 °C could reach up to 72 °C, depending on the nominal power and processing time.

According to the thermal histories shown in Figure 4, greater ΔT values are associated with lower working temperatures. The thermosonication treatment at 60 °C exhibited lower differences between the working temperature and maximum temperature of the whey beverages. The temperature can affect physical properties such as saturation vapor pressure, surface tension, sound velocity, and continuous phase’s viscosity, which is mainly composed of water. Our results suggested that the increase in temperature gradually decreased the acoustic cavitation intensity since nominal power increase did not promote the same response on the maximum temperature for each working temperature that was evaluated. We selected the working temperature of 60 °C for studying the impact of thermosonication treatments because in this condition the whey beverages reached the greater maximum temperature (84 °C), employing a nominal power of 300 W. Thus, our objective was to understand what would be the role of the dairy system and prebiotic ingredients to carry and protect the lipophilic bioactive compounds from fennel under high mechanical stress and moderate thermal conditions.

### 3.2. Technological Properties

#### 3.2.1. Droplet Size Distribution and Microstructure

Table 2 presents the impact of the thermosonication treatments and prebiotic ingredients on the droplet size distribution of the whey beverages according to their Sauter mean diameter ($D_{3,2}$) and diameters of cumulative volume. Nominal power and beverage formulation had a significant effect on $D_{3,2}$ values (*p*-value = 0.002). The increase in nominal power from 100 W to 300 W linearly reduced $D_{3,2}$ values. On the other hand, the enrichment of the whey beverages with FOS and XOS also reduced the Sauter mean diameter (*p*-value < 0.001). However, for the standard beverage, the formulation without prebiotic carbohydrates, the increase in nominal power increased the $D_{3,2}$ values.

**Figure 5:**
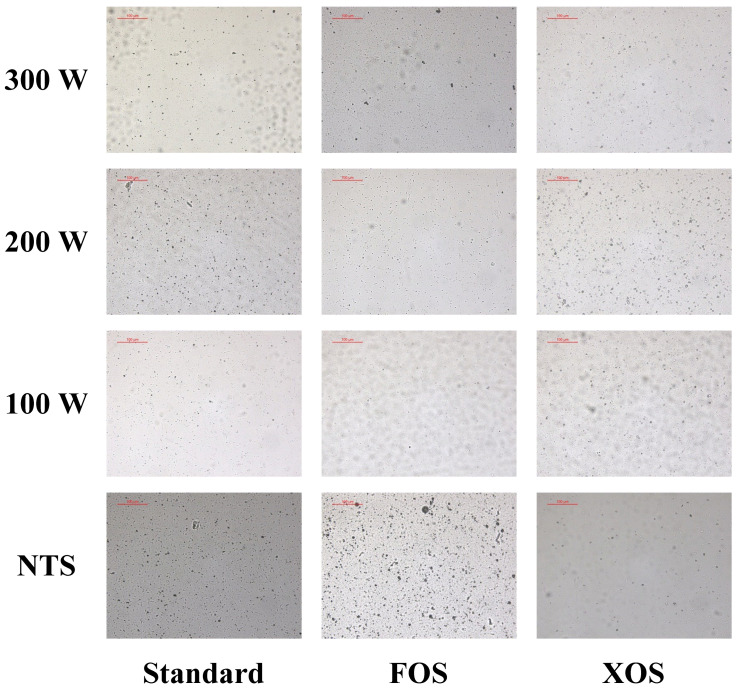
Impact of the thermosonication treatments and dietary fibers on the microstructure of the whey beverages. NTS: Non-thermosonicated. FOS: fructooligosaccharides. XOS: xylooligosaccharides.

The whey beverages evaluated in this study are complex systems designed to carry the fennel seed extract. Figure 5 shows the microstructure of the fennel-based whey beverages before and after thermosonication treatments. The microscopic images corroborated the observed results of droplet size distribution. The whey beverages evaluated in this study are colloidal systems. Whey proteins were used as emulsifier agents for the lipophilic compounds of the fennel extract obtained using the supercritical CO_2_ extraction technique. Thus, the reduction or increase in the $D_{3,2}$ values refers to size modulation of a fennel extract droplet. The main constituents of the continuous phase are water and soluble ingredients, which may not be seen in the optical micrographs.

The collapse of cavitation microbubbles due to the application of the acoustic field in the whey beverage is responsible for reducing $D_{3,2}$ values. High-energy shear stress can be applied from the collapse of microbubbles into the sonicated liquid medium, contributing to its homogenization and size modulation of the dispersed phase. Likewise, the physicochemical characteristics of the ingredients and their interactions have a strong impact on emulsion size distribution.

The addition of FOS and XOS contributed to the formation of micelles that were more resistant to the high-energy treatments. Likewise, other authors have also demonstrated improvements in technological properties in products enriched with prebiotic dietary fibers [43,44,45].

A protein–carbohydrate interaction by a glycation process through a non-enzymatic reaction has been recognized as a potential modification for improving the technological properties of proteins [46,47]. In this study, the Maillard reaction promoted a covalent bond between the whey proteins and FOS/XOS. Whey protein–FOS/XOS conjugates were produced during the emulsification step since it was assisted by heat (Figure 2). After that, the glycation process may also have occurred during thermosonication treatments due to moderate heat processing (Figure 4) because in both steps the heating process may have favored the occurrence of the Maillard reaction. Wang et al. [48] produced whey protein isolate (WPI)–inulin conjugates in aqueous solutions after subjecting them to wet-heat treatment at 70 °C for 2 to 6 h. The emulsifying properties of WPI were enhanced after its glycation with inulin. Therefore, the possible formation of whey protein–FOS/XOS conjugates likely improved the performance of the whey beverage for emulsifying the fennel extract, according to the results shown in Table 2. For the standard beverage, the increase in nominal power in the thermosonication treatments did not contribute to the reduction in $D_{3,2}$ values. On the other hand, the whey beverages enriched with FOS and XOS had their $D_{3,2}$ values reduced from approximately 1.0 to 0.7 µm at the same process conditions (*p*-value = 0.002). The droplet size modulation is not an exclusive function of the amount of energy provided to the colloidal system. Standard, FOS, and XOS beverages received the same amount of energy. However, the droplet size distribution of the standard beverage was negatively impacted by the increase in acoustic energy, as we can observe in Table 2. For this whey beverage, a re-coalescence phenomenon known as overprocessing was observed. The application of a high amount of energy favored the re-coalescence of the oil droplets, increasing the $D_{3,2}$ values (*p*-value = 0.002) and $d_{10}$, $d_{50}$, and $d_{90}$ values (*p* value ≤ 0.011).

#### 3.2.2. Kinetic Stability

**Figure 6:**
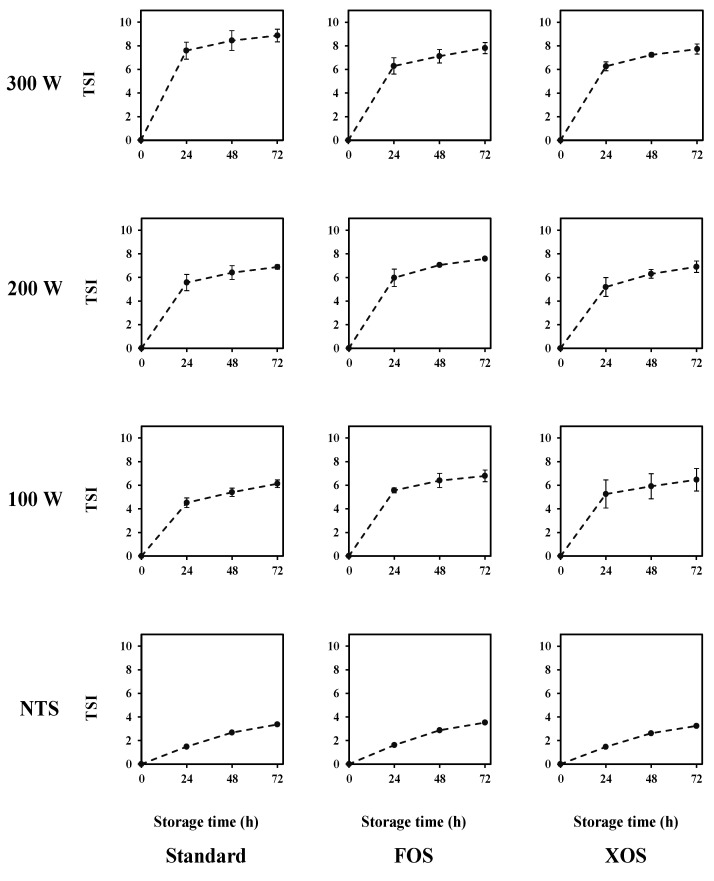
Impact of the thermosonication treatments and dietary fibers on the kinetic stability of the whey beverages. TSI: Turbiscan Stability Index. NTS: Non-thermosonicated. FOS: fructooligosaccharides. XOS: xylooligosaccharides.

The influence of nominal power and prebiotic dietary fibers on the kinetic stability of the whey beverages is presented in Figure 6. Turbiscan Stability Index (TSI) values were used for comparing the kinetic stability of the samples during their cold storage at 4 °C for 72 h. TSI values were obtained from the sum of all destabilization processes that occur in the measuring cell. Thus, the higher the TSI values, the greater the whey beverage destabilization. The maintenance of colloidal stability is an important technological property for dairy beverages. In this study, the kinetic stability strongly impacts functional activity because the whey beverages are carrying systems for the supercritical fennel extract.

**Figure 7:**
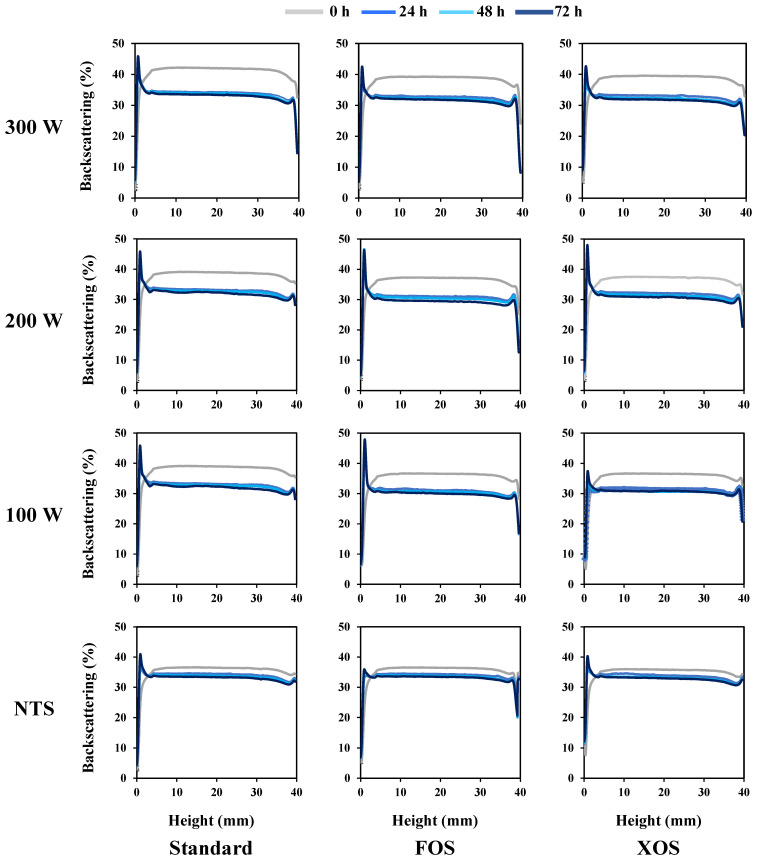
Impact of the thermosonication treatments and dietary fibers on the backscattering profile of the whey beverages. NTS: Non-thermosonicated. FOS: fructooligosaccharides. XOS: xylooligosaccharides.

**Figure 8:**
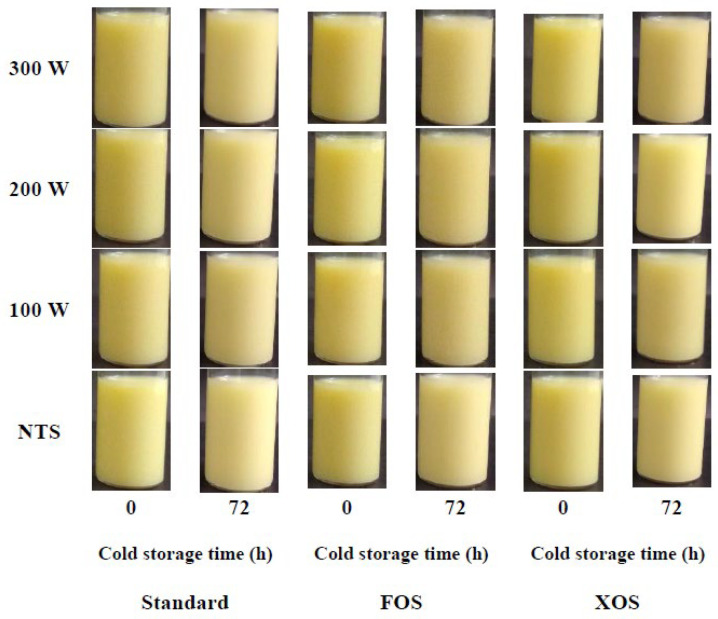
Impact of the thermosonication treatments and dietary fibers on the visual appearance of the whey beverages. FOS: fructooligosaccharides. XOS: xylooligosaccharides.

According to the results exhibited in Figure 6, the beverage supplementation with FOS and XOS did not affect the kinetic stability of the fennel-based whey. Both formulations presented similar behaviors for the evolution of TSI values during their cold storage. However, thermosonication treatments, for all nominal powers evaluated, increased the TSI values. A similar trend was observed for all treatments at the same nominal power, but the increase in nominal power for the same formulation presented a slight increase in the TSI values over the cold storage time. A similar trend was verified for the results of the backscattering (BS) profile of the samples (Figure 7). The BS (%) values are presented from 0 to 40 mm. For all samples at the different cold storage times, the BS (%) values were constant along tube length, indicating a uniform droplet size distribution. Both whey beverages had their BS (%) values decreased after 24 h. However, after that, the BS (%) values remained the same until 72 h. BS values can be correlated to the droplet size. For droplets higher than the incident wavelength (880 nm), BS values are inversely proportional to the droplet size [49]. Thermosonication treatments increased the initial BS (%) values, but after the storage time (equilibrium time), all samples presented a similar BS (%) value. The visual appearance of the fennel-based whey beverages right after their manufacturing and after their cold storage for 72 h are presented in the Figure 8. The pictures confirm the kinetic stability of the samples according to the results obtained by the multiple light-scattering technique.

#### 3.2.3. Color

Another important quality characteristic for dairy beverages is associated with their color attributes. Table 3 presents the effects of the thermosonication treatments and enrichment with dietary fibers on the instrumental color parameters, browning index, and color changes. The beverage supplementation with prebiotic ingredients decreased $L^{*}$ values (*p*-value = 0.018). However, nominal power did not influence the luminosity of the samples (*p*-value = 0.827). A similar behavior was verified for color changes (Δ*E*); only the FOS and XOS additions modified the instrumental color parameters (*p*-value = 0.021).

Δ*E* values indicate the overall difference between each treatment compared to non-thermosonicated beverages. However, the low Δ*E* values observed (≤2.11) indicate that the changes were indiscernible to the human eye (Figure 8). The browning index ($BI^{*}$) was influenced by both variables. XOS addition increased more $BI^{*}$ values than FOS (*p*-value = 0.003). The increase in nominal power increased $BI^{*}$ values (*p*-value < 0.001) due to more intense moderate thermal treatment. The combined effect of the external heat source and acoustic energy on the temperature increase of the whey beverages favored the Maillard reaction, contributing to the increase in the $BI^{*}$ values. These results are in agreement with that observed for the possible formation of whey protein–FOS/XOS conjugates. A similar trend was verified between the improvement in the emulsification performance of the whey beverages and their browning due to the Maillard reaction, which may have enhanced the technological properties of the whey proteins by means of their glycation with the prebiotic carbohydrates.

### 3.3. Chemical and Functional Properties

#### 3.3.1. Phenolic Compounds

**Figure 9:**
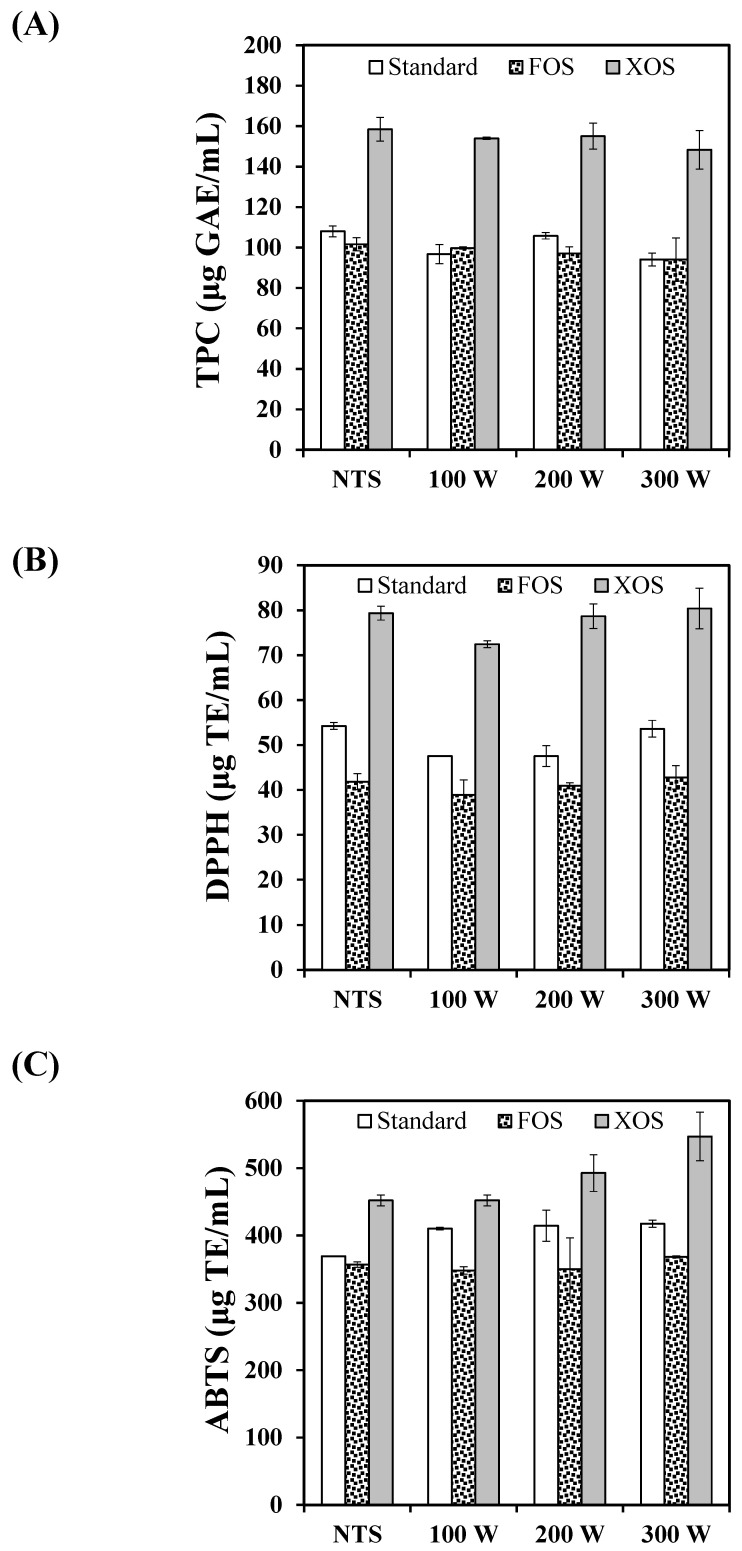
Impact of the thermosonication treatments and dietary fibers on the chemical and functional quality of the whey beverages. (**A**) TPC; (**B**) DPPH; (**C**) ABTS.

Figure 9A presents the impact of the thermosonication treatments and prebiotic ingredients on the total phenolic content (TPC). For all formulations, thermosonication did not reduce the TPC (*p*-value = 0.143). On the other hand, the enrichment with FOS and XOS changed the TPC of the fennel-based whey beverage (*p*-value < 0.001). For the non-thermosonicated samples, the whey beverage supplemented with XOS presented greater TPC values, 159 ± 6 µg GAE/mL. The standard formulation and the beverage enriched with FOS presented 108 ± 7 µg GAE/mL and 102 ± 3 µg GAE/mL, respectively. 

The XOS addition increased the TPC values compared to the standard formulation due to the phenolic compounds of the prebiotic ingredient. The manufacturing processes for transforming lignocellulosic biomass (sugarcane bagasse, corncob, wheat straw, and others) into XOS are generally performed in two stages. First, the xylan is extracted by one or a combination of pretreatments and then the xylan is hydrolyzed using xylanolytic enzymes [50]. Phenolic acids such as ferulic and *p*-coumaric may be used for the arabinose esterification [51]. In this way, the residual contents of these phenolic compounds can be found in commercial samples of XOS, which probably increased the TPC of the whey beverages enriched with XOS. 

The TPC maintenance after high-energy processes employing high mechanical stress and moderate heat treatments demonstrated the good performance of the whey-based colloidal systems as promising carrying matrices for the supercritical fennel extracts. Whey beverages were able to carry and protect the phenolic compounds during the thermosonication treatments with or without prebiotic ingredients. Bannikova et al. [52] microencapsulated polyphenols and xylooligosaccharides from oat bran in whey protein–maltodextrin complex coacervates. The high encapsulation efficiency of the phenolic compounds of 95.3% was obtained using a whey protein concentrate and maltodextrin ratio of 60:40.

#### 3.3.2. In Vitro Antioxidant Capacity

The fennel-based whey beverages’ antioxidant capacity was examined for their scavenging effect on the DPPH^•^ and ABTS^•+^ free radical activities (Figure 9B,C, respectively). For the DPPH^•^ and ABTS^•+^ assays, thermosonication treatments had a significant effect on the antioxidant capacity of the beverages (*p*-value = 0.008 and *p*-value = 0.005, respectively). The enrichment of the whey beverages with FOS and XOS also significantly affected both in vitro antioxidant capacity assays (*p*-value < 0.001).

Regarding the DPPH^•^ results for the standard beverage, the thermosonication treatments applying 100 W and 200 W promoted a slight decrease in the antioxidant capacity. However, the thermosonication with 300 W presented the same antioxidant capacity as the non-thermosonicated standard sample. For the beverage supplemented with XOS, only the thermosonication treatment with 100 W reduced the antioxidant capacity regarding other treatments. On the other hand, FOS-enriched beverages presented the same antioxidant capacity for all treatments. Despite the significant differences detected by the ANOVA test, the results demonstrated that the whey beverages’ antioxidant capacity, as measured by the DPPH^•^ assay, was preserved after the thermosonication treatments at 60 °C from 100 W to 300 W. Similar to the TPC results, the XOS-supplemented beverage presented a better performance on antioxidant capacity due to the phenolic acids used during the manufacturing of XOS. Concerning the ABTS^•+^ assays, the thermosonication treatments increased the antioxidant capacity of the standard beverage and did not affect the beverage enriched with FOS. The XOS-enriched beverage had its antioxidant capacity increased by the thermosonication treatments with 200 W and 300 W. Again, the ingredient XOS potentialized the antioxidant capacity due to its residual content of phenolic acids.

The fennel-based whey beverages are complex dairy systems and their supplementation with prebiotic fibers makes them even more complex in terms of their composition. In addition to the complexity of the colloidal systems examined in this study, the sonication combined with heat treatment may promote various chemical modifications in the macromolecules, such as whey proteins and oligosaccharides, and phenolic compounds. Therefore, a detailed explanation for the modulation of the antioxidant capacity regarding thermosonication treatments and different formulations is a big challenge. 

## 4. Conclusions

Fennel-based whey beverages were manufactured using low-frequency ultrasound for carrying lipophilic fennel extract obtained by supercritical CO_2_ extraction. The enrichment of the beverages with FOS and XOS did not affect their kinetic stability. However, the addition of prebiotic fibers contributed to reducing the mean droplet size due to the formation of whey protein–FOS/XOS conjugates. The high-energy processes employing high mechanical stress and moderate heat treatments did not promote color changes discernible to the human eye. On the other hand, thermosonication treatments reduced the kinetic stability of the whey beverages. According to the results for phenolic compounds, whey beverages presented a high performance as a carrying system for lipophilic bioactive compounds from fennel seeds. Overall, the colloidal dairy systems preserved the antioxidant capacity of the fennel seed extract, regardless of the thermosonication process intensity.

## Figures and Tables

**Table 1:** Formulation of the fennel-based whey beverages.

Whey Beverage	Fennel Extract (g/100 g)	Whey Powder (g/100 g)	Skimmed Milk Powder (g/100 g)	FOS (g/100 g)	XOS (g/100 g)	Deionized Water (g/100 g)
Standard	1	10	5	-	-	84
FOS	1	10	5	3	-	81
XOS	1	10	5	-	3	81

FOS: fructooligosaccharides; XOS: xylooligosaccharides.

**Table 2:** Impact of the thermosonication treatments and prebiotic dietary fibers (FOS and XOS) on the mean diameter ($D_{3,2}$) and cumulative diameters ($d_{10}$, $d_{50}$, and $d_{90}$) of the fennel-based whey beverages.

Ultrasonic Power	Whey Beverage	$D_{3,2}\;(µm)$	$d_{10}\;(µm)$	$d_{50}\;(µm)$	$d_{90}\;(µm)$
NTS	Standard	1.06 ± 0.04	0.477 ± 0.005	1.93 ± 0.3	10.1 ± 0.7
	FOS	1.05 ± 0.04	0.48 ± 0.01	1.73 ± 0.2	8.00 ± 0.05
	XOS	1.02 ± 0.01	0.473 ± 0.003	1.73 ± 0.1	8.30 ± 0.1
100 W	Standard	1.02 ± 0.03	0.47 ± 0.01	1.51 ± 0.6	8.8 ± 0.2
	FOS	0.97 ± 0.01	0.438 ± 0.003	1.82 ± 0.1	9.48 ± 0.03
	XOS	0.98 ± 0.02	0.44 ± 0.01	1.95 ± 0.1	8.6 ± 0.1
200 W	Standard	1.17 ± 0.04	0.49 ± 0.01	2.39 ± 0.1	12.3 ± 0.1
	FOS	0.76 ± 0.06	0.41 ± 0.02	0.67 ± 0.1	6.4 ± 0.6
	XOS	0.81 ± 0.03	0.417 ± 0.002	0.72 ± 0.03	6.8 ± 0.5
300 W	Standard	1.17 ± 0.07	0.49 ± 0.01	2.60 ± 0.3	13 ±1
	FOS	0.73 ± 0.03	0.40 ± 0.01	0.64 ± 0.02	6.2 ± 0.6
	XOS	0.72 ± 0.02	0.40 ± 0.01	0.64 ± 0.01	5.9 ± 0.2

NTS: Non-thermosonicated; FOS: fructooligosaccharides; XOS: xylooligosaccharides.

**Table 3:** Impact of the thermosonication treatments and dietary fibers (FOS and XOS) on the pH values, color parameters, and browning index of the fennel-based whey beverages.

Ultrasonic Power	Whey Beverage	pH	$L^{*}$	$a^{*}$	$b^{*}$	$C^{*}$	$H^{*\;}$	$BI^{*\;}$	ΔE
NTS	Standard	6.40 ± <0.01	63.8 ± 0.4	−4.84 ± 0.04	14.5 ± 0.1	15.3 ± 0.1	108.5 ± 0.3	19.0 ± 0.4	−
	FOS	6.37 ± 0.03	64.4 ± 0.9	−4.92 ± 0.08	14.7 ± 0.5	15.5 ± 0.4	108.5 ± 0.8	19.2 ± 0.7	−
	XOS	6.40 ± 0.01	63.6 ± 0.7	−4.89 ± 0.05	14.9 ± 0.2	15.7 ± 0.2	108.158 ± 0.003	19.8 ± 0.5	−
100 W	Standard	6.33 ± 0.03	63.7 ± 0.9	−4.9 ± 0.1	14.7 ± 0.2	15.5 ± 0.2	108.3 ± 0.2	19.5 ± 0.6	0.7 ± 0.2
	FOS	6.3 ± 0.1	63.2 ± 0.2	−4.83 ± 0.04	14.71 ± 0.07	15.48 ± 0.05	108.2 ± 0.2	19.7 ± 0.1	1.3 ± 0.2
	XOS	6.39 ± 0.02	63 ± 1	−4.71 ± 0.04	14.9 ± 0.1	15.6 ± 0.1	107.53 ± <0.01	20.4 ± 0.1	1.1 ± 0.8
200 W	Standard	6.33 ± 0.05	63.2 ± 0.4	−4.74 ± 0.06	15.1 ± 0.3	15.8 ± 0.2	107.5 ± 0.5	20.5 ± 0.7	0.8 ± 0.4
	FOS	6.36 ± <0.01	63.2 ± 0.8	−4.7 ± 0.1	15.2 ± 0.3	15.9 ± 0.2	107.2 ± 0.7	20.7 ± 0.4	1.5 ± 0.4
	XOS	6.3 ± 0.2	62.56 ± 0.02	−4.5 ± 0.1	15.6 ± 0.2	16.2 ± 0.2	106.2 ± 0.2	22.0 ± 0.2	1.74 ± 0.05
300 W	Standard	6.32 ± 0.06	63.93 ± 0.04	−4.50 ± 0.08	15.5 ± 0.1	16.2 ± 0.1	106.1 ± 0.4	21.4 ± 0.4	1.1 ± 0.2
	FOS	6.32 ± 0.01	63.3 ± 0.7	−4.4 ± 0.2	15.9 ± 0.2	16.5 ± 0.2	105.6 ± 0.4	22.4 ± 0.5	1.8 ± 0.5
	XOS	6.3 ± 0.1	61.8 ± 0.6	−4.17 ± 0.04	15.5 ± 0.4	16.1 ± 0.4	105.1 ± 0.2	22.7 ± 0.5	2.1 ± 0.4

NTS: Non−thermosonicated. FOS: fructooligosaccharides. XOS: xylooligosaccharides. $L^{*}$ represents the lightness with values from 0 (black) to 100 (white); positive values of $a^{*}$ are red and negative values are green; positive values of $b^{*}$ are yellow and negative values are blue; $C^{*}$ represents chromaticity; $H^{*\;}$ represents the hue of the color with values from 0° (red) to 270° (blue); $BI^{*\;}$is the browning index; and ΔE represents color difference.

## Data Availability

The data presented in this study are available in article.
